# Rethinking our strategies to develop disease-modifying therapies for Parkinson's: Time for combination therapies?

**DOI:** 10.1177/1877718X261434686

**Published:** 2026-04-21

**Authors:** Paloma Fernandez, Rachel M Hughes, Helen Matthews, Joy Duffen, Radi Haloub, Anthony H Schapira, Simon RW Stott

**Affiliations:** 1 Cure Parkinson's, London, UK; 2 University College London, London, UK

**Keywords:** Parkinson's disease, disease-modifying therapies, clinical trials, combination therapies

## Abstract

Despite significant advances in our understanding of Parkinson's disease (PD) over the last three decades, no disease-modifying therapies (DMTs) have been identified. There is considerable effort focused on this goal, but the challenges in achieving it are illustrated by the limited number of DMTs advancing to late-stage Phase 3 clinical testing (see annual *Parkinson's Disease Drug Therapies in the Clinical Trial Pipeline* publications). The lack of progress towards slowing the disease is further highlighted by the recent disappointing results from high profile, Phase 2/3 clinical trials for DMTs. In this commentary, we would like to suggest an alternative strategy for identifying DMTs. Specifically, we propose the evaluation of rationally designed combination therapies that target multiple PD etiological factors.

DMTs, defined as therapies that slow, stop or reverse disease progression, have become a critical component in the treatment of many medical conditions, including neurodegenerative diseases like multiple sclerosis. However, they remain elusive for PD. By early 2025, several high-profile Phase 2 trials for PD-DMT had delivered disappointing results, leading to the developmental pause or discontinuation of drugs previously considered promising innovative assets, such as risvodetinib (Inhibikase),^1^ minzasolmin (UCB/Novartis)^2^ and the Phase 3 clinical trial of the GLP-1 receptor agonist exenatide.^3^ While there may be additional work on some of these agents, these setbacks, coupled with the slow development of DMT candidates to later-stage clinical testing,^4^ reflect the challenges in developing effective DMTs for PD. They also highlight problems such as the disconnect between preclinical models and human studies, the lack of definitive biomarkers for disease progression, and the reliance on clinical endpoints requiring lengthy follow-up. Moreover, current approaches tend to ignore the likelihood of multiple different biological disease mechanisms active within the patient population.

**Figure 1.**
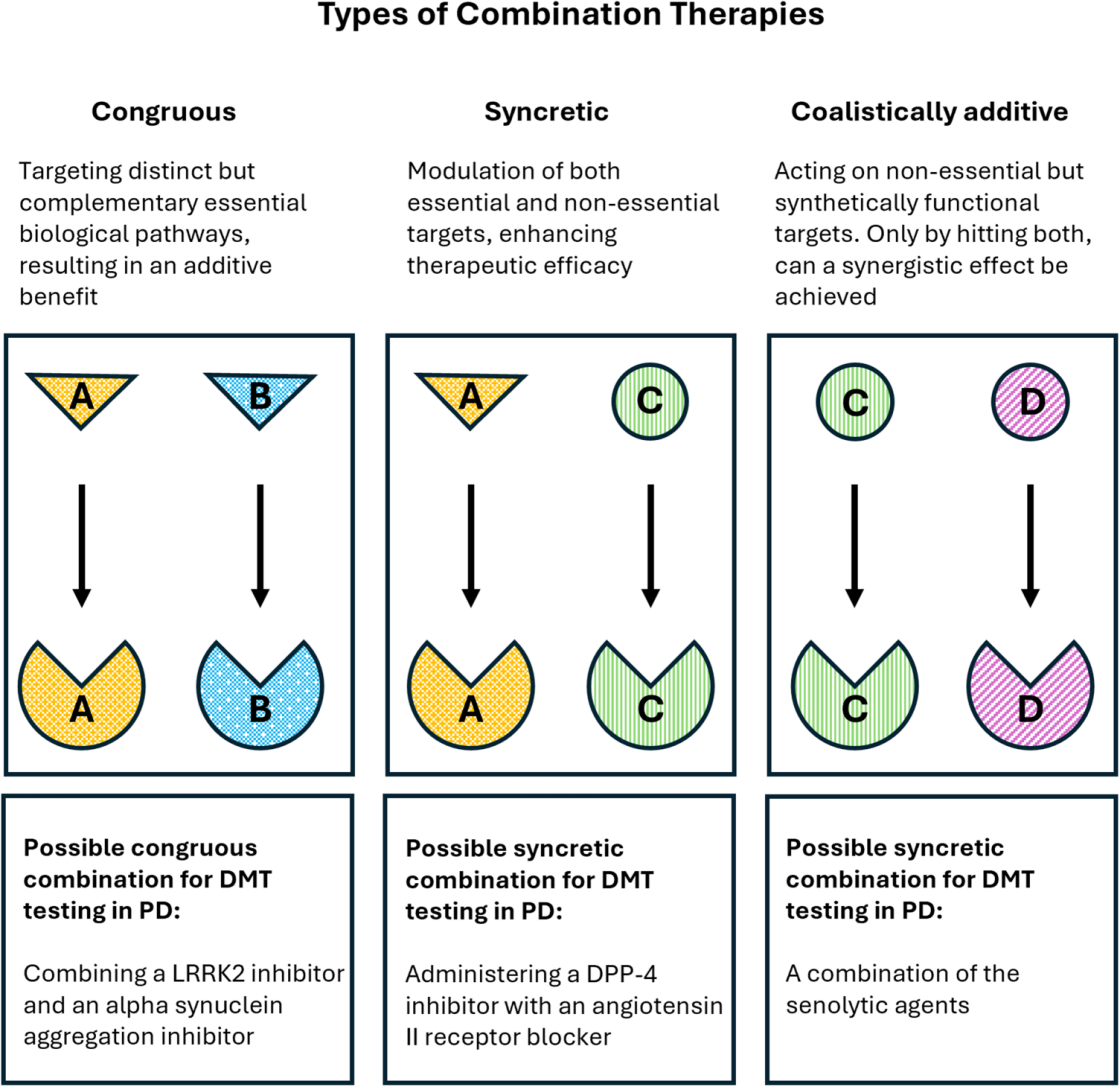
Types of combination therapies. Congruous: two agents that target essential biological pathways; syncretic: one agent targets an essential pathways and the other agent targets a non-essential pathway; coalistically additive: two agents that act on non-essential pathways. Figure adapted with permission from Tyer & Wright.^5^

Thus, the continued emphasis on testing monotherapies targeting single pathways as potential PD-DMTs, may be limiting progress. One alternative could be the evaluation of rationally designed combination therapies.

Since the first recorded trial of the joint use of streptomycin and para-aminosalicylic acid in tuberculosis in 1950, there have been different approaches applied to combinatorial therapies which are now employed across disease indications (particularly oncology, infectious diseases and cardiovascular disease). Pharmacologically, combinations can be refer to (Figure 1):^5^

- *Congruous*: agents that target distinct but complementary essential biological pathways, resulting in an additive benefit. For example, a combination of the calcium channel blocker amlodipine and the angiotensin II receptor blocker valsartan, where the dual action of vasodilation by the former and prevention of vasoconstriction by the latter yields a more effective reduction in blood pressure, particularly beneficial in managing severe hypertension.
- *Syncretic*: agents that lead to a synergistic effect by simultaneously modulating both essential and non-essential targets, thereby enhancing therapeutic efficacy. For example, a combination of the antibiotic amoxicillin and the beta-lactamase inhibitor clavulanic acid, where the latter inactivates bacterial enzymes (beta-lactamases) that break down amoxicillin, thus preserving antibacterial effect and preventing antibiotic resistance.
- *Coalistically additive*: agents that act on non-essential but synthetically functional targets, where the therapeutic effect is observed only by acting on both targets. Although clinical examples are limited, this approach has been explored in oncology leveraging the concept of synthetic lethality. For example, the combined inhibition of PARP1, which is involved in base excision DNA repair, and RAD52, which participates in backup DNA repair pathways, induces synthetic lethality in BRCA-deficient cancer cells.^6^ These cells are particularly vulnerable to DNA damage due to the loss of *BRCA1/2* tumor suppressor genes, which are essential for high-fidelity homologous recombination repair.

Combination therapy is not a new concept in PD. The standard of care, carbidopa-levodopa (e.g., Sinemet), is a classic example of a syncretic combination. This symptomatic treatment is based on the mix of a peripheral DOPA decarboxylase inhibitor (carbidopa) and the amino-acid that is the precursor to dopamine (levodopa). There is also an ongoing clinical trial investigating a combination as a DMT. The Australian Parkinson's Mission Study 2, a Phase 2 multi-arm trial platform, is evaluating the effect of ambroxol and doxycycline both separately and in combination.^7^ The former agent is an expectorant that has been shown to enhance waste clearance in cells (via the lysosomal enzyme glucocerebrosidase)^8^ while the latter has been demonstrated to prevent alpha-synuclein aggregation.^9^

Looking forward, potential combination strategies for testing disease-modification in PD could be:

- *Congruous:* LRKK2 inhibitor with an alpha synuclein aggregation inhibitor. Expression of both proteins has been identified as universal hallmarks in PD pathology and potential drug targets.
- *Syncretic:* DPP-4 inhibitor (i.e., omarigliptin) with an angiotensin II receptor blocker (i.e., telmisartan). *AGTR1* (the gene encoding the angiotensin II receptor) has been reported to be elevated in the substantia nigra of people with PD and blocking it has shown neuroprotective effects in PD models.^10^ On the other hand, DPP-4 degrades the incretin hormones GLP-1 and GIP, as well as other proteins that could be beneficial in PD. The two pathways are also bi-directionally connected^11^ and epidemiological data support their protective role in PD.^12,13^
- *Coalistically additive:* combination of the senolytic agents dasatinib (tyrosine-kinase inhibitor) and quercetin (flavonoid) which has been reported to effectively eliminate senescent cells in preclinical models of Alzheimer's, and is now being clinically tested.^14^ In PD, senescent cell markers have been reported to be present in the putamen and within astrocytes of the substantia nigra. Notably, the removal of these senescent cells in both *in vitro* and *in vivo* models reduces the impact of neurotoxin-induced pathology in PD.^15^

While arguments could be made both in support of and against these examples, there is growing consensus in the research community that a combination of agents on top of standard of care will ultimately be required for slowing PD progression.^16^ Despite this, the field remains largely focused on evaluating experimental agents as monotherapies. This approach assumes that a ‘silver bullet’ molecule can effectively overcome the complex nature of PD and has yet to deliver meaningful advances. We accept the limitations of our suggestion, particularly in combining patented compounds still under investigation. However, we propose that the use of repurposed drugs offer an important alternative option for early testing of combination therapies in PD.

Our proposed adjustment to the current approach for identifying PD-DMTs is not a radical shift, but a pragmatic step to improve our prospects of success. Accordingly, while we will continue evaluating the potential efficacy of monotherapies, we will also aim to explore the potential synergistic benefits of combination therapies. Moreover, as we move into a new age of multi-arm, multi-stage clinical trial platforms that provide the option of comparing multiple treatment arms with a single placebo arm, the opportunity presents to assess combinations of agents in PD in a more cost-effective manner than traditional approaches. These initiatives will also help in building our understanding of biomarker dynamics, enabling more precise selection of patient subgroups for targeted interventions, and ultimately increasing the likelihood of success.

We encourage the research community to embrace this proposed approach and join us in proactively identifying drug combinations with the potential to effectively modify the course of PD.
